# A National Study of Social Media, Television, Radio, and Internet Usage of Adults by Sexual Orientation and Smoking Status: Implications for Campaign Design

**DOI:** 10.3390/ijerph14040450

**Published:** 2017-04-21

**Authors:** Andrew B. Seidenberg, Catherine L. Jo, Kurt M. Ribisl, Joseph G. L. Lee, Francisco O. Buchting, Yoonsang Kim, Sherry L. Emery

**Affiliations:** 1 Department of Health Behavior, Gillings School of Global Public Health, University of North Carolina at Chapel Hill, Chapel Hill, NC 27599, USA; catherinejo@alumni.duke.edu (C.L.J.); kurt_ribisl@unc.edu (K.M.R.); LEEJOSE14@ECU.EDU (J.G.L.L.); 2 Lineberger Comprehensive Cancer Center, University of North Carolina at Chapel Hill, Chapel Hill, NC 27599, USA; 3 Department of Health Education and Promotion, College of Health and Human Performance, East Carolina University, Greenville, NC 27858, USA; 4 Horizons Foundation, San Francisco, CA 96766, USA; franciscobuchting@outlook.com; 5 Health Media Collaboratory, National Opinion Research Center at the University of Chicago, Chicago, IL 60602, USA; Kim-Yoonsang@norc.org (Y.K.); Emery-Sherry@norc.org (S.L.E.)

**Keywords:** smoking, LGBT, media

## Abstract

*Background*: Smoking rates among lesbian, gay, and bisexual (LGB) people significantly exceed that of heterosexuals. Media interventions are an important part of tobacco control efforts, but limited information is available on LGB people’s media use. *Methods*: A nationally representative sample of 12,900 U.S. adults completed an online questionnaire assessing media use, smoking status, and demographic information. Multivariable logistic regression was used to assess relationships between media use with sexual orientation and smoking status. *Results*: A total of 590 (4.6%) respondents identified as LGB, of which 29% were smokers. Regardless of sexual orientation and smoking status, the Internet was the most popular media channel used, followed by television and radio. LGB respondents had significantly greater odds of having accounts on social media websites, accessing Facebook daily, and being a frequent Internet user, compared to heterosexual respondents. Similar media use was found between smokers and non-smokers, but smokers had greater odds of being frequent television viewers and frequent Internet users, compared to non-smokers. *Conclusions*: Compared to heterosexuals, LGB respondents reported greater use of the Internet, especially social media. Media campaigns targeting LGB populations can maximize reach by utilizing social media alongside traditional media channels.

## 1. Introduction

Cigarette smoking rates among lesbian, gay, and bisexual (LGB) people significantly exceed the rates of heterosexual (straight) people [1,2]. The 2013 U.S. National Health Interview Survey found that smoking prevalence among LGB adults (27%) was more than 50% greater than that of straight adults (18%) [3]. Across LGB subpopulations, cigarette smoking is highest among bisexuals (37%), followed by gays (29%) and lesbians (27%) [4].

The identification of smoking disparities among LGB people has resulted in the development of interventions targeting these communities, including tobacco control media campaigns [5]. While many of the existing media campaigns have historically been small and have been driven by LGB organizations (e.g., Mautner Project’s “Delicious Lesbian Kisses” campaign [6]) or state level programs (e.g., the California Tobacco Control Program [7]), there are newer larger national examples. The Centers for Disease Control’s “Tips from Former Smokers” campaign included testimonials from former LGB smokers and ran ads on an LGB-oriented cable network [8]. The Food and Drug Administration’s “This Free Life” campaign is a $36 million educational campaign designed to prevent and reduce the consequences of tobacco use among LGB young adults running in select TV markets and online [9].

Tobacco control mass media campaigns have been shown to reduce smoking initiation among youth [10] and encourage smoking cessation among adults [11]. The Centers for Disease Control and Prevention’s Best Practices for Comprehensive Tobacco Control Programs recommends the use of mass media campaigns [12]. However, growing evidence shows that, while mass media campaigns have an overall impact on population health, they may not reduce disparities; in fact, they may exacerbate them [13,14]. For instance, Niederdeppe and colleagues conducted a systematic review on the effectiveness of mass media campaigns on promoting smoking cessation within populations of low socioeconomic status and found disparities in the efficacy of media campaigns across levels of socioeconomic advantage. Specifically, the authors report that differences in campaign exposure, motivational response, and opportunity to sustain quitting long-term each contribute to a widening of cigarette smoking disparities by socioeconomic advantage [13]. In a separate review, Hill et al. found evidence that non-targeted smoking cessation programs increase inequalities because such programs are more effective among individuals of higher socio-economic standing [14]. One pro-equity strategy is to ensure targeted sub-campaigns to specific populations delivered through media channels that reach the specific population [15]. Thus, information about the media use habits of LGB populations is needed for developing campaigns to reach this audience.

Despite increasing interest in LGB-targeted tobacco control media campaigns, little is known regarding how best to reach LGB populations. For instance, previous systematic reviews have found no peer-reviewed research on media use habits of LGB communities in relation to smoking status [5,16]. Understanding LGB media use patterns would help ensure that LGB populations are meaningfully exposed to campaign messages [13]. Therefore, we surveyed a nationally representative US sample to assess the media use habits of LGB smokers and non-smokers. Media use habits of heterosexual smokers and non-smokers were also assessed for comparison. 

## 2. Methods

This study includes data from a nationally representative sample of U.S. adults, and is part of a larger study assessing tobacco-related content encountered across different media platforms. These data were collected in 2013 from the GfK Group’s (GfK) KnowledgePanel, an online probability-based panel [17]. GfK recruited KnowledgePanel members by using random digit dialing and address-based sampling. This sampling frame combined with weighting ensured a representative result of the U.S. population, accounting for listed and unlisted telephone numbers; households that were telephone, non-telephone, or cell-phone-only; and households with and without Internet access. Post-stratification weights were constructed incorporating demographic characteristics (age, gender, education, income, etc.) that are correlated with sexual orientation [4], helping to minimize potential bias for comparisons between LGB and non-LGB populations. If needed, respondents (*n* = 859) were provided with a computer and Internet access to ensure all qualified respondents could complete the online questionnaire. Of the 34,097 KnowledgePanel members sampled, 61% (*n* = 20,907) underwent screening, of which 65% (*n* = 13,531) were determined to be eligible. Eligible respondents were 18 years of age or older, living in the U.S., and had not completed a KnowledgePanel survey in the current week (no more than one survey is assigned per week to each panel member). In addition, current cigarette smokers were over-sampled.

Ninety-seven percent of eligible panel members (*n* = 13,144) completed the online questionnaire. After removing observations with missing responses or responses set to missing for a sexual orientation item (*n* = 244), the final analytic sample was 12,900. The University of Illinois at Chicago’s institutional review board approved this study (IRB Protocol #2011-0470).

### 2.1. Measures

The independent variables were sexual orientation and cigarette smoking status. Respondents were asked “Do you consider yourself to be …” and sexual orientation response categories included heterosexual, lesbian, gay, bisexual, or other. Respondents who selected “other” were able to provide further detail in a free-form text field, which was reviewed and recoded as either one of the other four categories or missing. To increase power, respondents indicating that they were lesbian, gay, or bisexual were combined into a single category, hereafter referred to as LGB. Current cigarette smoking was defined as having smoked at least 100 cigarettes in lifetime and currently smoking every day or some days. 

We assessed the use of devices that serve as access points to the Internet, as well as the use of specific social media websites. Type of device owned (i.e., laptop computer/netbook, smartphone, or tablet) was assessed to understand the ways in which respondents might access media. We also investigated if respondents had accounts on the following social media websites: Facebook, Twitter, LinkedIn, Google+, and YouTube. A dichotomous variable was created for having accounts on ≥3 of these sites. Among those reporting having an account on Facebook and Twitter, respondents were asked about their frequency of use of these websites. Responses were dichotomized into daily users or not. 

Further, respondents were asked how many hours in a typical week they used three major media channels: television (i.e., network broadcast, cable, or satellite), Internet, and radio (“including satellite and talk radio, like Sirius XM, or streaming music”). Respondents who indicated watching 11 or more hours of television per week and spending four or more hours on the Internet were coded as being frequent television users and frequent Internet users, respectively. Those who reported listening to two or more hours of radio per week were coded as being frequent radio users. Frequent use approximately represented the highest media consuming tertile based on the distribution of responses for each media type. 

We controlled for the potential confounding effects of various demographic and socioeconomic variables that could be independently associated with sexual orientation, smoking status, or media consumption. Demographic variables included age, gender (male or female), race/ethnicity, and geographic region, while socioeconomic variables included employment status and years of education. Transgender identity was assessed by an item separate from the gender item. Employment status was composed of three categories: unemployed—in labor force (i.e., looking for work or temporarily laid off); unemployed—not in labor force (i.e., retired or disabled); and employed (i.e., paid or self-employment).

### 2.2. Data Analysis

Stata version 14 was used for all analyses. Weighting adjustments were made for all analyses to adjust for any known deviations from probability sampling during sample selection. Weighted estimates (%) for device ownership, having a social media account, and frequency of media (television, Internet, radio/streaming music, and social media) use were calculated among LGB and heterosexual respondents, and among smokers and non-smokers. Design-based F-tests were used to test for significant differences between groups. In addition, weighted multivariable logistic regression models were used to compare odds of device ownership, having a social media account, and media use among LGB and heterosexual respondents (referent group = heterosexuals). A binary independent variable for smoking status (smoker vs. nonsmoker) was included in all models to assess differences in odds of all outcomes by smoking status (referent group = nonsmokers). To test whether differences in media use between LGB and heterosexuals varied by smoking status, interaction terms were added to all models (sexual orientation × smoking status). An interaction term (gender × sexual orientation) was added to assess whether gender moderated the relationship between sexual orientation and media use. Interaction terms were added independently. 

Due to conducting tests for the many outcome variables, we controlled for the false discovery rate (FDR) in all models at the level 5% by adjusting thresholds to determine statistical significance by using the method by Benjamini and Yekutieli [18].

## 3. Results

A total of 13,144 respondents completed the cross-sectional online survey. Among these, 98% (*n* = 12,900) of respondents identified as either heterosexual or LGB, with 590 (4.6%) respondents identifying as being LGB. Of the LGB respondents, 243 (41.2% of LGB respondents) identified as gay, 118 (20.0%) identified as lesbian, and 229 (38.8%) identified as bisexual. We did not exclude transgender respondents, but do not report separate results due to the small number (*n* = 72) of transgender respondents (i.e., transgender respondents with non-missing responses to gender and sexual orientation were included). Twenty-nine percent of LGB respondents were current smokers, compared to 17.8% of heterosexual respondents. Table 1 describes the demographic characteristics of the four groups of respondents: heterosexual non-smokers, heterosexual smokers, LGB non-smokers, and LGB smokers.

### 3.1. Device Ownership

Laptop/netbook was the most commonly owned device among all groups, followed by smartphone and tablet, respectively. A greater percentage of LGB respondents reported owning a laptop, smartphone, and tablet; however, odds of ownership for these devices did not differ by sexual orientation in adjusted models. Smokers had significantly lower odds of owning tablet (OR = 0.65; 95% CI = 0.56, 0.75), compared to nonsmokers. While smokers had lower odds of smartphone ownership (OR = 0.84; 95% CI = 0.72, 0.97) compared to non-smokers, this difference was not statistically significant after controlling for FDR (Table 2).

### 3.2. Social Media

Table 3 presents the odds ratios for having social media accounts. Compared with heterosexual respondents, a greater percentage of LGB respondents reported having a social media presence on Facebook, Twitter, LinkedIn, Google+, and YouTube. Further, in adjusted models, LGB respondents had significantly greater odds of having an account on Facebook (OR = 2.00; 95% CI = 1.48, 2.70), Twitter (OR = 1.84; 95% CI = 1.37, 2.47), Google+ (OR = 2.11; 95% CI = 1.55, 2.87), or YouTube (OR = 2.19; 95% CI = 1.63, 2.94), compared with heterosexual respondents. LGB respondents also had significantly greater odds of having an account on ≥3 social media websites (OR = 2.38; 95% CI = 1.80, 3.16), compared with heterosexual respondents. 

Few differences were found comparing social media presence between smokers and non-smokers (Table 3). While a greater percentage of smokers reported having a Facebook account compared to nonsmokers, odds of having a Facebook profile did not differ significantly by smoking status in the adjusted model (OR = 1.15; 95% CI = 0.99, 1.33). Odds of having a social media presence on Twitter, Google+, or YouTube, or having a presence on ≥3 social media websites, did not significantly differ by smoking status. 

### 3.3. Frequent/Daily Media Use

Over 98% of respondents reported accessing the Internet weekly, compared to 92% and 79% reporting any weekly television watching and any weekly radio/streaming music listening, respectively. LGB respondents had significantly greater odds of reporting frequent weekly Internet use, compared with heterosexual respondents (OR = 1.89; 95% CI = 1.45, 2.46). In contrast, no significant differences were found between LGB and heterosexual respondents for reporting frequent weekly television watching (OR = 0.95; 95% CI = 0.73, 1.24) or frequent weekly radio/streaming music listening (OR = 0.92; 95% CI = 0.70, 1.21). Further, LGB respondents had significantly greater odds of reported daily Facebook use, compared to heterosexual respondents (OR = 1.63; 95% CI = 1.27, 2.11). However, daily Twitter use did not differ significantly by sexual orientation (OR = 1.45; 95% CI = 0.89, 2.37; Table 4).

Analyses comparing frequent and daily media use among smokers and non-smokers is also presented in Table 4. Compared to non-smokers, cigarette smokers reported significantly greater odds of frequent television watching (OR = 1.33; 95% CI = 1.15, 1.54) and Internet use (OR = 1.38; 95% CI = 1.18, 1.62). In contrast, no significant difference in odds was found between smokers and non-smokers for being a frequent radio listener (OR = 1.11; 95% CI = 0.95, 1.28) or accessing Facebook (OR = 1.10; 95% CI = 0.95, 1.27) or Twitter daily (OR = 1.01; 95% CI = 0.71, 1.46).

All interaction terms (sexual orientation × smoking status, and sexual orientation × gender) were found to be non-significant (*p* > 0.05) and thus were removed from all models (all reported results are from models that did not contain interaction terms).

## 4. Discussion

Compared to heterosexuals, LGB respondents reported greater use of online media, especially social media. LGB respondents reported significantly greater odds of having numerous social media accounts and accessing Facebook daily, compared to heterosexuals. LGB respondents also reported significantly greater odds of frequent Internet use, compared to heterosexuals. No significant differences were found for having social media presence between smokers and non-smokers; however, smokers had significantly greater odds of being frequent television and Internet users. These findings suggest that tobacco control mass media campaigns should consider utilizing web-based media, including social media, when targeting LGB populations. While Internet usage was the most frequently consumed media type, about one in every seven LGB respondents reported having none of the social media accounts we assessed, and high proportions of LGB respondents reported weekly television (>90%) and radio (75%) use. Therefore, media campaigns should also include traditional media channels.

While this is the first peer-reviewed publication to our knowledge to report that LGB smokers and non-smokers are frequent users of social media (compared to heterosexuals), this finding is consistent with several non-peer-reviewed reports. For instance, a 2013 survey conducted by the Pew Research Center found that 80% of lesbian, gay, bisexual, and transgender (LGBT) Americans reported using social networking websites, and 55% reported meeting new LGBT friends online [19]. Similarly, a 2010 national survey conducted by Harris Interactive found that 54% of gay and lesbian adults read online blogs compared to 40% of heterosexual adults, and 73% had Facebook accounts compared to 65% of heterosexuals [20]. Such reports are similar to the data presented here, showing greater use of Facebook among LGB respondents. 

The reasons for these differences in online media usage are unclear. It is plausible that LGB people may use online websites to find friends, partners, and communities in ways that are not always available in their physical communities [21,22]. Research with LGBT youth, for example, documents the importance of finding LGBT communities online in building resilience against discrimination and in coming out [23,24]. In addition, LGBT youth are more likely to use the Internet to explore and express their sexuality than non-LGBT youth [22]. As a small proportion of the population, LGB people may be driven online to find friends and romantic partners, especially in rural areas [21,25]. Further, several social media platforms have been specifically designed for members of the LGBT community, including MOOVZ, HORNET, Distinc.tt, Transtastic, TrevorSPACE, and a variety of dating and hook-up apps (e.g., Grindr and Scruff) targeted at specific segments of the LGBT community. Social media can also serve as a major source of news and social support for LGBTQ users. For instance, @TwitterOpen, @ItGetsBetter, @TheAdvocateMag, @outmagazine, @huffpostqueer, @glaad, @QueerHistoryQDM, and @QueerStoriesQDM news and community accounts each have over 100 K followers on Twitter.

An alternative explanation may be the combination of the spatial distribution of LGB people toward more urban centers (both regionally and nationally) [26] and the potentially more rapid diffusion of technology access and uptake. Indeed, although untested to our knowledge in relation to social media, Florida [27] speculates that desirable locations for LGB people that promote tolerance also include greater exposure to a creative class of people who may be earlier adopters of social media innovations. Insomuch as LGB people, as a population, migrate toward more open, accepting, and potentially creative places [28], they may be more exposed to technological innovations. However, further investigation is necessary.

Campaigns utilizing online platforms to reach audiences have several advantages. First, online campaigns can be highly targeted to a desired population. For instance, Facebook advertising can target audiences by location, age, interests, and other factors [29]. Secondly, online media allows audiences to actively engage with content, and share content, which may enhance the effectiveness of campaigns. Thirdly, online media is less expensive than traditional media (e.g., television and radio). A recent analysis assessing awareness of a national tobacco educational campaign found that television ads generated higher levels of awareness, but online videos were more cost-effective [30].

Content promoting tobacco use has been well documented on social media. For instance, Tobacco Company marketing has appeared on Facebook, Twitter, and YouTube [31,32,33]. Moreover, user-generated (non-industry) content encouraging tobacco use has been described [34,35]. Frequent social media use may expose the LGB community to tobacco-promoting content online, which may be contributing to high smoking prevalence.

LGB communities’ media outlets are known to have limited content promoting tobacco cessation and a substantial amount of content marketing tobacco products or showing them in a positive light [36,37]. In a Chicago, IL, community survey, LGB respondents were more likely to have seen anti-tobacco messaging in the non-LGBT specific media [38]. The only other evidence about electronic LGBT news sources comes from a content analysis of popular LGBT news blogs, which found just 105 tobacco control-related posts over a nine-year period [39]. Social media clearly offers a largely untapped opportunity to promote tobacco control messages. Further, smoking cessation interventions have utilized social media components with some success [40].

There are important limitations to this study. While the sampling frame covered households without computer and Internet access, this was a web-based survey and may overestimate Internet usage. Secondly, the weighted sample may not be representative of LGB populations. Further, while we controlled for several demographic and socioeconomic characteristics that could potentially confound the relationships between sexual orientation and media use, confounding due to variables not measured or not included in these models may still remain. Finally, due to small sample sizes, the analyses did not differentiate between gay men, lesbians, male bisexuals, and female bisexuals. Instead, these groups were all combined into a single sexual minority group, which limits understanding of media use among sexual minorities by gender. 

Current trends reveal that fewer younger Americans are watching television or purchasing a cable subscription; instead, they are viewing television programming and other video content through the Internet (e.g., Netflix, Amazon Prime, and HBO Go) [41]. In this rapidly changing media environment, public health practitioners must adapt to the media use habits of their audience. This is especially relevant to reaching LGB smokers and non-smokers due to their greater use of the Internet and social media. Further, given the importance of social networks, narratives, and interpersonal communication in initiating and succeeding in a cessation attempt, social media represents an important channel for media campaigns. Campaign developers must also acknowledge that social media campaigns will not reach all LGB smokers, so supplementation with traditional channels is needed. 

## 5. Conclusions

To reduce tobacco-promoting content, some countries, like Australia, have implemented comprehensive bans on tobacco product advertising, including on the Internet and other electronic media [42]. However, given the global nature of the Internet, such bans alone are not sufficient to prevent exposure to tobacco product advertising, particularly on social media [43]. Counter marketing campaigns from the tobacco control community are essential as well.

This study found LGB respondents reporting greater use of the Internet and social media. Social media and other Internet-based interventions have been used to promote a variety of healthy lifestyles, including diet, exercise, and smoking cessation [40,44,45,46]. Interventions promoting smoking cessation have utilized Facebook and Twitter, both popular among LGB populations, and have been shown to be acceptable and efficacious in the short term [40,46]. Thus, media campaigns targeting LGB populations can maximize reach by utilizing social media alongside traditional media channels. 

Additional research is needed to elucidate what messages may work best for LGB media campaigns, and no studies have tested LGB-specific messages in social media channels. While these data suggest that online media (especially social media) may be an effective strategy for reaching LGB populations, additional research is needed to understand how LGB individuals engage with media and advertising online [5]. Further, identifying which social media platforms are best for reaching segments of the LGB community with higher smoking rates, or at risk for smoking, is essential for maximizing the impact of online media campaigns. Additionally, because youth represent an important audience for many tobacco control media campaigns, further research is needed to understand the media use habits of LGB adolescents. In summary, these findings suggest that tobacco control media campaigns and other health behavior change interventions relevant to LGB populations may increase their reach by utilizing Internet and social media platforms.

## Figures and Tables

**Table 1:** Weighted % (unweighted sample size) demographic characteristics for heterosexual non-smokers, heterosexual smokers, LGB non-smokers, and LGB smokers.

Characteristic	Heterosexual Non-Smoker (*n* = 10,121)	Heterosexual Smoker (*n* = 2189)	LGB Non-Smoker (*n* = 418)	LGB Smoker (*n* = 172)	*p*-Value
**Gender**					
Female	51.9 (*n* = 5422)	54.0 (*n* = 1342)	39.6 (*n* = 172)	39.3 (*n* = 82)	<0.001
**Age**					
18–24	8.8 (*n* = 553)	6.6 (*n* = 121)	13.8 (*n* = 36)	7.9 (*n* = 14)	
25–44	33.6 (*n* = 2516)	37.0 (*n* = 593)	33.7 (*n* = 126)	40.9 (*n* = 69)	
45–64	37.0 (*n* = 4145)	46.1 (*n* = 1110)	45.3 (*n* = 201)	46.4 (*n* = 72)	
65+	20.6 (*n* = 2907)	10.2 (*n* = 365)	7.2 (*n* = 55)	4.9 (*n* = 17)	0.213
**Race**					
White	69.5 (*n* = 8164)	70.9 (*n* = 1739)	64.1 (*n* = 314)	65.1 (*n* = 128)	
Black	10.9 (*n* = 705)	12.4 (*n* = 192)	10.0 (*n* = 30)	12.8 (*n* = 20)	
Hispanic	12.7 (*n* = 689)	11.7 (*n* = 138)	16.2 (*n* = 43)	14.1 (*n* = 13)	
Other or Multi-race	6.9 (*n* = 563)	4.9 (*n* = 120)	9.8 (*n* = 31)	7.9 (*n* = 11)	<0.001
**Employment**					
Unemployed—In Labor Force	17.0 (*n* = 1308)	21.8 (*n* = 391)	21.2 (*n* = 68)	15.9 (*n* = 23)	
Unemployed—Not in Labor Force	25.3 (*n* = 3284)	24.5 (*n* = 662)	17.9 (*n* = 94)	16.0 (*n* = 34)	
Employed	57.7 (*n* = 5529)	53.7 (*n* = 1136)	61.0 (*n* = 256)	68.1 (*n* = 115)	<0.001
**Education**					
Some High School or Less	5.6 (*n* = 306)	11.4 (*n* = 137)	4.5 (*n* = 9)	6.9 (*n* = 8)	
High School Graduate	33.3 (*n* = 2003)	43.6 (*n* = 598)	20.9 (*n* = 45)	30.1 (*n* = 34)	
Some College	31.3 (*n* = 3126)	33.3 (*n* = 938)	33.5 (*n* = 112)	49.5 (*n* = 85)	
Bachelor Degree	17.7 (*n* = 2781)	8.2 (*n* = 357)	21.3 (*n* = 138)	7.3 (*n* = 30)	
Masters Degree or higher	12.1 (*n* = 1905)	3.6 (*n* = 159)	19.8 (*n* = 114)	6.3 (*n* = 15)	<0.001
**Region**					
Northeast	18.5 (*n* = 1761)	16.9 (*n* = 391)	20.3 (*n* = 71)	23.5 (*n* = 32)	
Midwest	22.1 (*n* = 2629)	26.4 (*n* = 627)	20.0 (*n* = 106)	22.9 (*n* = 44)	
South	36.5 (*n* = 3475)	38.8 (*n* = 745)	30.5 (*n* = 120)	37.4 (*n* = 65)	
West	22.9 (*n* = 2256)	17.9 (*n* = 426)	29.2 (*n* = 121)	16.2 (*n* = 31)	0.007

Notes: LGB = lesbian, gay, or bisexual.

**Table 2:** Multivariable logistic regression results for device ownership.

Variable	Laptop or Netbook		Cell (Smart)		Tablet	
	aOR	*p*-Value	aOR	*p*-Value	aOR	*p*-Value
**LGB vs. Heterosexual**						
Heterosexual	ref		ref		ref	
LGB	1.07 (0.78, 1.48)	0.6660	1.19 (0.90, 1.58)	0.2170	1.10 (0.85, 1.42)	0.4778
**Smoker vs. Nonsmoker**						
Nonsmoker	ref		ref		ref	
Smoker	0.90 (0.76, 1.05)	0.1823	0.84 (0.72, 0.97)	0.0175	0.65 (0.56, 0.75)	<0.0001 *
**Gender**						
Male	ref		ref		ref	
Female	0.94 (0.83, 1.07)	0.3658	0.94 (0.83, 1.05)	0.268	1.18 (1.05, 1.32)	0.0038
**Age**						
18–24	ref		ref		ref	
25–44	0.79 (0.58, 1.07)	0.1313	0.68 (0.52, 0.88)	0.0039	1.41 (1.09, 1.82)	0.0082
45–64	0.55 (0.41, 0.74)	0.0001 *	0.27 (0.21, 0.35)	<0.0001 *	1.20 (0.93, 1.54)	0.1586
65+	0.36 (0.26, 0.50)	<0.0001 *	0.15 (0.11, 0.20)	<0.0001 *	1.00 (0.75, 1.33)	0.9922
**Race**						
White	ref		ref		ref	
Black	0.73 (0.59, 0.91)	0.0050	1.30 (1.06, 1.59)	0.0126	0.75 (0.61, 0.93)	0.0072
Hispanic	1.00 (0.79, 1.25)	0.9708	1.56 (1.27, 1.92)	<0.0001 *	1.02 (0.84, 1.24)	0.8328
Other or Multi	1.15 (0.87, 1.51)	0.3235	1.42 (1.11, 1.82)	0.0061	1.15 (0.90, 1.46)	0.2566
**Education**						
Some High School or Less	ref		ref		ref	
High School Graduate	1.25 (0.95, 1.65)	0.1075	1.58 (1.17, 2.14)	0.0028 *	1.40 (1.02, 1.91)	0.0359
Some College	1.80 (1.37, 2.36)	<0.0001 *	2.66 (1.97, 3.59)	<0.0001 *	1.91 (1.40, 2.60)	<0.0001 *
Bachelor Degree	2.82 (2.11, 3.78)	<0.0001 *	3.23 (2.37, 4.40)	<0.0001 *	2.70 (1.97, 3.71)	<0.0001 *
Master’s Degree or Higher	3.36 (2.46, 4.58)	<0.0001 *	3.78 (2.74, 5.21)	<0.0001 *	3.26 (2.34, 4.52)	<0.0001 *
**Employment**						
Unemployed-In Labor Force	ref		ref		ref	
Unemployed-Not in Labor Force	0.93 (0.75, 1.16)	0.5369	0.79 (0.64, 0.98)	0.0296	0.74 (0.60, 0.91)	0.0042
Employed	1.13 (0.94, 1.36)	0.2011	1.59 (1.34, 1.87)	<0.0001 *	1.14 (0.97, 1.35)	0.1122
**Region**						
Northeast	ref		ref		ref	
Midwest	0.71 (0.59, 0.85)	0.0003 *	0.91 (0.76, 1.07)	0.2524	0.89 (0.76, 1.06)	0.1848
South	0.86 (0.72, 1.03)	0.1020	1.20 (1.02, 1.41)	0.0254	1.00 (0.86, 1.18)	0.9609
West	0.72 (0.59, 0.87)	0.0009 *	1.13 (0.94, 1.35)	0.1844	0.92 (0.77, 1.09)	0.3279
Number of observations	12,894		12,883		12,892	
F-Test	21.67		55.54		20.22	
*p*-value	<0.0001 *		<0.0001 *		<0.0001 *	
F-adj mean res goodness of fit	1.365		0.246		0.607	
*p*-value	0.198		0.988		0.792	

Notes: * *p*-value < FDR-adjusted threshold.

**Table 3:** Multivariable logistic regression results for having a social media profile/account.

Variable	Facebook		Twitter		LinkedIn		Google+		YouTube		≥3 Social Media Accounts	
	aOR	*p*-Value	aOR	*p*-Value	aOR	*p*-Value	aOR	*p*-Value	aOR	*p*-Value	aOR	*p*-Value
**LGB vs. Heterosexual**												
Heterosexual	ref		ref		ref		ref					
LGB	2.00 (1.48, 2.70)	<0.0001 *	1.84 (1.37, 2.47)	<0.0001 *	1.45 (1.09, 1.91)	0.0095	2.11 (1.55, 2.87)	<0.0001 *	2.19 (1.63, 2.94)	<0.0001 *	2.38 (1.80, 3.16)	<0.0001 *
**Smoker vs. Nonsmoker**												
Nonsmoker	ref		ref		ref		ref		ref		ref	
Smoker	1.15 (0.99, 1.33)	0.0769	1.07 (0.87, 1.30)	0.5335	0.78 (0.65, 0.95)	0.0143	1.01 (0.82, 1.25)	0.8993	0.92 (0.74, 1.13)	0.4204	0.93 (0.76, 1.14)	0.5047
**Gender**												
Male	ref		ref		ref		ref		ref		ref	
Female	1.76 (1.57, 1.97)	<0.0001 *	1.03 (0.89, 1.20)	0.6834	0.92 (0.81, 1.05)	0.2388	0.84 (0.72, 0.99)	0.0369	0.60 (0.51, 0.71)	<0.0001 *	0.84 (.72, 0.97)	0.0203
**Age**												
18–24	ref		ref		ref		ref		ref		ref	
25–44	0.70 (0.49, 0.73)	0.0191	0.43 (0.33, 0.56)	<0.0001 *	1.56 (1.11, 2.18)	0.0098	0.83 (0.64, 1.11)	0.2124	0.44 (0.34, 0.57)	<0.0001 *	0.60 (0.46, 0.78)	0.0001 *
45–64	0.39 (0.29, 0.51)	<0.0001 *	0.22 (0.17, 0.28)	<0.0001 *	1.71 (1.22, 2.40)	0.0019 *	0.34 (0.25, 0.45)	<0.0001 *	0.15 (0.11, 0.20)	<0.0001 *	0.27 (0.21, 0.35)	<0.0001 *
65+	0.25 (0.18, 0.34)	<0.0001 *	0.11 (0.07, 0.16)	<0.0001 *	1.28 (0.87, 1.89)	0.2081	028 (0.19, 0.42)	<0.0001 *	0.06 (0.04, 0.10)	<0.0001 *	0.17 (0.11, 0.24)	<0.0001 *
**Race**												
White	ref		ref		ref		ref		ref		ref	
Black	0.60 (0.49, 0.73)	<0.0001 *	1.31 (1.01, 1.69)	0.0417	0.82 (0.63, 1.05)	0.1097	1.73 (1.34, 2.24)	<0.0001 *	1.79 (1.29, 2.32)	<0.0001 *	1.31 (1.02, 1.69)	0.0335
Hispanic	0.83 (0.67, 1.02)	0.0784	1.13 (0.88, 1.45)	0.3237	0.74 (0.58, 0.94)	0.0134	1.23 (0.95, 1.59)	0.1091	1.27 (0.99, 1.63)	0.0594	1.09 (0.86, 1.40)	0.4724
Other or Multi	0.98 (0.76, 1.27)	0.8749	1.29 (0.95, 1.75)	0.1065	1.01 (0.77, 1.33)	0.957	1.84 (1.37, 2.48)	0.0001 *	1.82 (1.35, 2.43)	0.0001 *	1.65 (1.24, 2.18)	0.0005 *
**Education**												
Some HS or Less	ref		ref		ref		ref		ref		ref	
HS Graduate	1.07 (0.81, 1.40)	0.6327	0.93 (0.62, 1.41)	0.7476	1.83 (0.94, 3.55)	0.0751	0.72 (0.49, 1.07)	0.102	1.24 (0.82, 1.87)	0.3119	1.01 (0.65, 1.56)	0.9784
Some College	1.45 (1.10, 1.90)	0.0076	1.59 (1.07, 2.37)	0.0222	5.00 (2.62, 9.52)	<0.0001 *	1.18 (0.81, 1.73)	0.3791	1.97 (1.31, 2.95)	0.0010 *	2.23 (1.46, 3.40)	0.0002 *
Bachelor Degree	1.38 (1.04, 1.83)	0.0265	2.22 (1.47, 3.34)	0.0001 *	11.98 (6.27, 22.91)	<0.0001 *	1.33 (0.89, 1.97)	0.1616	1.85 (1.21, 2.83)	0.0042	3.04 (1.97, 4.70)	<0.0001 *
Master’s Degree or Higher	1.39 (1.03, 1.87)	0.03	1.98 (1.28, 3.06)	0.0021 *	13.38 (6.95, 25.77)	<0.0001 *	1.28 (0.84, 1.95)	0.2515	1.71 (1.09, 2.69)	0.0191	2.85 (1.81, 4.50)	<0.0001 *
**Employment**												
Unemployed—In Labor Force	ref		ref		ref		ref		ref		ref	
Unemployed—Not in Labor Force	0.95 (0.77, 1.18)	0.6627	0.68 (0.49, 0.93)	0.0165	0.68 (0.51, 0.90)	0.008	0.79 (0.58, 1.09)	0.1493	0.59 (0.43, 0.81)	0.0010 *	0.65 (0.48, 0.87)	0.0047
Employed	1.10 (0.92, 1.32)	0.2912	0.92 (0.75, 1.14)	0.4452	1.48 (1.19, 1.83)	0.0004 *	0.84 (0.68, 1.05)	0.1236	0.70 (0.57, 0.86)	0.0007 *	0.89 (0.72, 1.09)	0.2627
**Region**												
Northeast	ref		ref		ref		ref		ref		ref	
Midwest	1.14 (0.97, 1.35)	0.1206	1.17 (0.93, 1.48)	0.1687	0.89 (0.73, 1.09)	0.2762	1.20 (0.93, 1.54)	0.1567	0.97 (0.76, 1.24)	0.801	0.98 (0.78, 1.24)	0.8709
South	1.27 (1.08, 1.49)	0.0033	1.04 (0.84, 1.29)	0.7133	0.90 (0.74, 1.09)	0.2714	1.14 (0.90, 1.45)	0.2776	0.94 (0.74, 1.19)	0.5902	1.00 (0.80, 1.24)	0.9969
West	1.21 (1.01, 1.46)	0.0394	0.90 (0.70, 1.16)	0.4212	1.11 (0.90, 1.37)	0.3346	1.36 (1.05, 1.76)	0.0215	1.14 (0.88, 1.47)	0.3154	0.95 (0.74, 1.21)	0.6625
Number of observations	12,900		12,900		12,900		12,900		12,900		12,900	
F-Test	24.34		25.04		39.35		17.53		33.26		26.82	
*p*-value	<0.0001 *		<0.0001 *		<0.0001 *		<0.0001 *		<0.0001 *		<0.0001 *	
F-adj mean res goodness of fit	1.008		0.332		0.394		1.185		0.607		0.775	
*p*-value	0.431		0.965		0.939		0.299		0.792		0.640	

Notes: * *p*-value < FDR-adjusted threshold.

**Table 4:** Multivariable logistic regression results for frequent and daily media use.

Variable	Frequent TV Watching		Frequent Internet Use		Frequent Radio/Streaming Music		Daily Facebook Use		Daily Twitter Use	
	aOR	*p*-Value	aOR	*p*-Value	aOR	*p*-Value	aOR	*p*-Value	aOR	*p*-Value
**LGB vs. Heterosexual**										
Heterosexual	ref		ref				ref		ref	
LGB	0.95 (0.73, 1.24)	0.6931	1.89 (1.45, 2.46)	<0.0001 *	0.92 (0.70, 1.21)	0.5356	1.63 (1.27, 2.11)	0.0002 *	1.45 (0.89, 2.37)	0.1331
**Smoker vs. Nonsmoker**										
Nonsmoker	ref		ref				ref		ref	
Smoker	1.33 (1.15, 1.54)	0.0001 *	1.38 (1.18, 1.62)	<0.0001 *	1.11 (0.95, 1.28)	0.19	1.10 (0.95, 1.27)	0.196	1.01 (0.71, 1.46)	0.9442
**Gender**										
Male	ref						ref		ref	
Female	0.82 (0.73, 0.92)	0.0006 *	1.11 (0.97, 1.26)	0.126	0.75 (0.67, 0.85)	<0.0001 *	1.80 (1.60, 2.03)	<0.0001 *	0.75 (0.57, 0.98)	0.0335
**Age**										
18–24	ref		ref				ref		ref	
25–44	1.88 (1.35, 2.62)	0.0002 *	0.57 (0.44, 0.73)	<0.0001 *	1.16 (0.89, 1.51)	0.2758	0.65 (0.51, 0.82)	0.0004 *	0.35 (0.24, 0.51)	<0.0001 *
45–64	4.61 (3.34, 6.36)	<0.0001 *	0.42 (0.33, 0.54)	<0.0001 *	1.43 (1.10, 1.84)	0.007	0.32 (0.25, 0.41)	<0.0001 *	0.12 (0.08, 0.19)	<0.0001 *
65+	4.69 (3.30, 6.67)	<0.0001 *	0.22 (0.16, 0.31)	<0.0001 *	1.25 (0.93, 1.69)	0.141	0.17 (0.12, 0.22)	<0.0001 *	0.05 (0.02, 0.13)	<0.0001 *
**Race**										
White	ref		ref				ref		ref	
Black	0.84 (0.68, 1.03)	0.0931	1.38 (1.11, 1.71)	0.0031	0.60 (0.48, 0.75)	<0.0001 *	0.46 (0.37, 0.58	<0.0001 *	1.82 (1.21, 2.74)	0.0043
Hispanic	0.77 (0.62, 0.96)	0.0216	1.23 (0.99, 1.53)	0.0674	0.91 (0.74, 1.13)	0.3996	0.74 (0.60, 0.92)	0.0053	1.62 (1.13, 2.34)	0.0095
Other or Multi	0.71 (0.55, 0.93)	0.0111	1.98 (1.53, 2.55)	<0.0001 *	0.61 (0.47, 0.79)	0.0002 *	0.71 (0.55, 0.91)	0.0081	0.96 (0.57, 1.64)	0.89
**Education**										
Some HS or Less	ref		ref				ref		ref	
HS Graduate	1.30 (0.98, 1.72)	0.0729	0.94 (0.70, 1.27)	0.6978	1.33 (0.96, 1.85)	0.0891	1.34 (1.00, 1.79)	0.0469	0.53 (0.28, 1.00)	0.0518
Some College	1.18 (0.89, 1.57)	0.2475	1.18 (0.88, 1.58)	0.274	1.65 (1.19, 2.29)	0.0026 *	1.51 (1.13, 2.01)	0.0047	0.90 (0.49, 1.66)	0.7338
Bachelor Degree	1.17 (0.88, 1.57)	0.2806	0.84 (0.61, 1.15)	0.2774	2.00 (1.43, 2.80)	<0.0001 *	1.37 (1.02, 1.85)	0.0386	1.42 (0.75, 2.68)	0.2855
Master’s Degree or Higher	0.91 (0.67, 1.23)	0.5461	0.74 (0.53, 1.04)	0.0867	2.05 (1.45, 2.89)	<0.0001 *	1.27 (0.93, 1.73)	0.1394	1.39 (0.69, 2.77)	0.3553
**Employment**										
Unemployed—In Labor Force	ref		ref				ref		ref	
Unemployed—Not in Labor Force	1.44 (1.17, 1.78)	0.0007 *	1.01 (0.80, 1.27)	0.938	0.90 (0.71, 1.13)	0.363	0.85 (0.69, 1.06)	0.1543	0.38 (0.19, 0.75)	0.0055
Employed	0.85 (0.71, 1.01)	0.069	0.54 (0.45, 0.65)	<0.0001 *	1.59 (1.33, 1.90)	<0.0001 *	0.97 (0.83, 1.15)	0.7555	0.73 (0.52, 1.03)	0.0776
**Region**										
Northeast	ref		ref				ref		ref	
Midwest	1.04 (0.88, 1.23)	0.6322	0.78 (0.64, 0.94)	0.011	0.96 (0.82, 1.14)	0.6623	1.04 (0.87, 1.23)	0.6696	1.30 (0.88, 1.93)	0.1927
South	0.85 (0.72, 1.00)	0.05	0.93 (0.78, 1.11)	0.4514	0.83 (0.70, 0.97)	0.0211	1.14 (0.96, 1.34)	0.1347	1.00 (0.69, 1.46)	0.997
West	0.89 (0.75, 1.07)	0.2129	0.83 (0.68, 1.02)	0.0822	0.96 (0.80, 1.15)	0.6657	0.96 (0.80, 1.16)	0.7044	1.05 (0.69, 1.60)	0.8285
Number of observations	12,838		12,774		12,900		12,900		12,900	
F-Test	27.28		18.37		14.07		30.4		14.38	
*p*-value	<0.0001 *		<0.0001 *		<0.0001 *		<0.0001 *		<0.0001 *	
F-adj mean res goodness of fit	0.82		1.169		0.763		1.949		0.179	
*p*-value	0.598		0.31		0.651		0.041		0.996	

Notes: Adjusted for gender, age, race/ethnicity, education, employment, region; Frequent TV = 11 or more hours of tv/week; Frequent Internet = 4 or more hours/weekday; Frequent Radio/Streaming Music = 2 or more hours/week; * *p*-value < FDR-adjusted significant level.
